# Laparoscopic evaluation and management of isolated gastric rupture in a boy after blunt abdominal injury

**DOI:** 10.11604/pamj.2017.27.173.12430

**Published:** 2017-07-05

**Authors:** Anastasiadis Kleanthis, Vasilis Mouravas, Vasilis Lampropoulos, Evgenia Babatseva, Ioannis Spyridakis

**Affiliations:** 1 Paediatric Surgeon, 2^nd^ Department of Paediatric Surgery, Aristotle University of Thessaloniki, General Hospital Papageorgiou, Thessaloniki, Greece; 2 Paediatrician, Neonatologist, 2^nd^ Department of Neonatal Intensive Care, Aristotle University of Thessaloniki, General Hospital Papageorgiou, Thessaloniki, Greece; 3 Assistant Professor, Chief of the 2^nd^ Department of Paediatric Surgery, Aristotle University of Thessaloniki, General Hospital Papageorgiou, Thessaloniki, Greece

**Keywords:** Gastric rupture, abdominal trauma, laparoscopic surgery, minimally invasive surgery, children, trauma

## Abstract

Blunt abdominal injury in children can be a significant diagnostic and therapeutic challenge. The extent and localization of organ damage cannot be always thoroughly investigated noninvasively and in spite of modern imaging techniques and a laparotomy may be necessary for diagnosis, even though it carries a significant morbidity. We present a rare case of isolated gastric rupture after blunt abdominal injury in a 12 year old boy that sustained a bicycle accident. He was hemodynamically stable, had signs of acute abdomen and axial tomography was inconclusive as of the site of visceral perforation. Definitive diagnosis and treatment were carried out laparoscopically with excellent results. Laparoscopic surgery in cases of blunt abdominal injury with gastric rupture can serve both as a diagnostic and therapeutic modality with the additional advantage of being less traumatic. The accumulation of relevant experience is mandatory in order to establish this modality in the diagnostic and therapeutic protocols.

## Introduction

Laparoscopic surgery (LS) is gradually substituting open surgery not just as an alternative operative technique but also in the planning of patient management in pediatric blunt abdominal injuries (BAI) [1, 2]. Our report of an unusual case of gastric rupture aims to widen the accumulating experience on this field.

## Patient and observation

A 12 years old boy was referred to our facility one hour after sustaining a BAI caused falling on the steering bar of his bicycle. He was hemodynamically stable and laboratory report was normal. He reported epigastric pain and his upper abdomen was tender, with guarding. The child was cooperative and did not carry any other major wounds. No pain could be elicited in the thorax, the pelvis, or other areas of the body. An IV line was established. A supine chest X-ray and a CT scan of the abdomen and pelvis with IV contrast were performed. Free intraabdominal air, especially in the upper abdomen and significant amount of free fluid in the right and left paracolic gutter and around the stomach with considerable soiling of the area were evident. A large amount of free fluid was present in the pelvis. Radiologists indicated the anterior gastric wall, the duodenum and the sigmoid colon as the most likely points of injury. No other pathology was apparent. A nasogastric tube was placed which revealed bloody gastric content.

**Figure 1:**
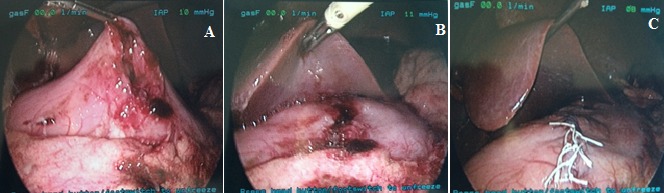
(A) gastric juice appears from below the liver; (B) right lobe of the liver is elevated and gastric trauma is visible; (C) gastric trauma is sutured

Since the child was hemodynamically stable, he was taken to the operating room for a laparoscopic abdominal exploration. The operation was performed with the use of a transumbical 12-mm trocar and a 10 mm 30° optic. Under vision, a 3mm trocar on the right epigastrium and a 5mm trocar on the left epigastrium were inserted. CO_2_ pneumoperitoneum at 10mmHg and 4L/min flow was established. Upon introducing the scope, a frothy liquid material was observed between the liver and the stomach (Figure 1 A). By lifting the right lobe, a 3cm long full thickness fissure on the anteriorsuperior gastric wall was evident (Figure 1B). Meticulous examination of the intraabdominal organs did not reveal any other injuries. Primary one layer closure of the rupture was performed with interrupted laparoscopic Ti-cron 2-0 sutures (Figure 1C). Air was insufflated through the NG tube and no leak was observed between the sutures. The abdominal cavity was evacuated from the liquid and rinsed properly. A thorough re-evaluation of the organs was performed once again and no other injury was found. The patient recovered from the operating table uneventfully. During his stay he received antibiotic treatment, PPI medication and TPN for 6 days. Intestinal motility resumed by the fourth day. He was discharged on the 8^th^ postoperative day. He was instructed to resume normal diet progressively. On follow up two months after surgery, he was found to be asymptomatic. Four months after surgery he was fully active.

## Discussion

Gastric rupture occurs in 70-90% of cases as a result of a motor vehicle accident (car, two wheeler, pedestrian) as the body accommodates the high energy of sudden velocity change into increased luminal pressure, causing gastric blowout [3]. Because this mechanism affects more or less all the intraabdominal organs, isolated traumatic gastric rupture after BAI is a rare condition and usually occurs in combination with other intraabdominal injuries, a factor which makes it more difficult to get to the diagnosis [4, 5]. The presence and severity of intraabdominal organ injuries is the main concern in children with BAI and in spite of great improvements in imaging technology, it is still often difficult to evaluate their extent, while significant morbidity is associated with missed or delayed diagnosis [1]. On the other hand, the golden standard of diagnostic laparotomy is not without risks, including a 20% morbidity rate, up to 5% mortality, and a 3% long-term risk for bowel obstruction [1]. In short, in cases with diagnostic dilemmas, two opposing pitfalls are considered by the physician: a laparotomy that will not prove therapeutic versus a therapeutic delay [3]. Although with a considerable time lag, compared to adults, diagnostic and therapeutic protocols that include LS have been proposed for childhood abdominal injuries [2]. The rate of a missed diagnosis of injury with laparoscopy in the adult literature is reported at 1%-3% [2]. LS has been successful in excluding or diagnosing injury and in completing therapeutic intervention in 65% of 200 patients with a mean age of 9.6-4.2 years, with a 0% missed injury rate [2]. Our case reinforces these statistics and increases the range of abdominal pathology with successful laparoscopic diagnosis and treatment. LS for gastric rupture in a child appears in literature only once before [6].

## Conclusion

Laparoscopy can enhance the strategic algorithms for BAI. It allows both the diagnosis and treatment of these occult injuries, while mitigating the concern about the morbidity of a laparotomy. More specifically, in gastric rupture, this approach is effective, allowing a thorough inspection of the abdomen, evacuation of the irritable and contaminated material and reconstruction of the gastric wall.

## Competing interests

The authors declare no competing interest.
